# Investigation of the Wear Performance of TiB_2_ Coated Cutting Tools during the Machining of Ti6Al4V Alloy

**DOI:** 10.3390/ma14112799

**Published:** 2021-05-24

**Authors:** Mohammad Shariful Islam Chowdhury, Bipasha Bose, German Fox-Rabinovich, Stephen Clarence Veldhuis

**Affiliations:** Department of Mechanical Engineering, McMaster Manufacturing Research Institute (MMRI), McMaster University, 1280 Main Street West, Hamilton, ON L8S4L7, Canada; boseb@mcmaster.ca (B.B.); gfox@mcmaster.ca (G.F.-R.); veldhu@mcmaster.ca (S.C.V.)

**Keywords:** rough turning, Ti6Al4V alloy, PVD coating, built-up edge (BUE), flank wear, tribo-films, magnetron sputtering

## Abstract

The machining of Ti6Al4V alloy, especially at low cutting speeds, is associated with strong Built-Up Edge (BUE) formation. The PVD coatings applied on cutting tools to machine such materials must have the necessary combination of properties to address such an underlying wear mechanism. The present work investigates and shows that TiB_2_ PVD coating can be designed to have certain mechanical properties and tribological characteristics that improve machining in cases where BUE formation is observed. Three TiB_2_ coatings were studied: one low hardness coating and two high hardness coatings with varied coating thicknesses. Wear performances for the various TiB_2_ coated carbide tools were evaluated while rough turning Ti6Al4V. Tool wear characteristics were evaluated using tool life studies and the 3D wear volume measurements of the worn surface. Chip morphology analyses were done to assess the in-situ tribological performance of the coatings. The micro-mechanical properties of the coatings were also studied in detail to co-relate with the coatings’ performances. The results obtained show that during the rough turning of Ti6Al4V alloy with intensive BUE formation, the harder TiB_2_ coatings performed worse, with coating delamination on the rake surface under operation, whereas the softer version of the coating exhibited significantly better tool life without significant coating failure.

## 1. Introduction

Titanium alloys possess an exceptional combination of mechanical and chemical properties (high ductility, good toughness, high-temperature strength, corrosion resistance, and a good strength-to-weight ratio) making them very popular in the aerospace and automobile industries [1,2,3,4,5]. However, they are very difficult to machine owing to certain unique characteristics like low thermal conductivity and a high chemical affinity towards tool materials. These result in high cutting temperatures during machining and built-up edge formation due to intensive adhesive interaction at the tool-chip interface, causing the carbide tools to wear rapidly. Consequently, to have a reasonably long tool life, low cutting speeds are typically used when machining titanium, sacrificing productivity.

For almost all machining applications, tool coatings have had significant success at reducing tool wear rates and improving productivity. However, when it comes to machining titanium, coatings have not achieved the same level of success. In many cases, coated cemented carbide tools perform worse than uncoated tools, especially during rough turning at low cutting speeds where the major phenomenon dictating tool performance is intensive BUE formation [6]. Many researchers have used PVD coated tools to improve tool performance when machining titanium alloys. However, most widely used PVD coatings, such as AlTiN, Al_2_O_3_, HfN, TiC, TiN, TiCN, TiN/TiC, TiN/TiC/TiN, and Al_2_O_3_/TiC, exhibit lower efficiency than uncoated tools [4,7,8]. This is due to how BUE formation instigates cohesive debonding and peeling of the coating layer under load [9,10,11]. Some researchers [12,13], however, have reported a minor improvement in tool performance with TiN, TiAlN, cBN+TiAlN, and TiN/TiCN/TiN coated tools.

Although PVD coated tools have had limited success with machining titanium, coating technology has advanced considerably over the years, and with the evolution of coating deposition technologies and a better understanding of the unique tribological requirements of machining titanium, new coating designs are being proposed [14]. Amongst others, PVD boride coatings have shown significant promise for machining titanium alloys [15]. TiB_2_ coatings have high thermal and chemical stability at room and elevated temperatures [15,16,17,18,19]. Such properties make TiB_2_ coatings very promising candidates for machining applications where BUE formation is observed. However, TiB_2_ coatings have high hardness and residual stresses making the coatings brittle with low substrate adhesion [16,17]. This is counterproductive for such applications. However, this issue can be resolved by varying coating deposition parameters and by using various deposition technologies like magnetron sputtering [19,20,21]. Recently, it has also been shown that TiB_2_ coatings have strong self-lubricating properties within the cutting zone [2]. These properties develop due to the ability of the coatings to form beneficial tribo-films through interaction with their environment [22]. For machining applications where BUE formation occurs, it is of the utmost importance to enhance the lubricity of the coating since it can minimize BUE formation and prevent coating detachment under load [9,23].

For the present paper, three TiB_2_ coatings deposited by magnetron sputtering were studied: two high hardness TiB_2_ coatings of varied thicknesses and one low hardness TiB_2_ coating. One of the main goals of this paper is to relate the wear performance of TiB_2_ coated carbide tools during the rough turning of Ti6Al4V to their mechanical and adaptive characteristics. The novelty of the present study lies in identifying the best performing TiB_2_ coating for machining applications where BUE formation is observed, and an in-depth analysis of the relevant properties which make it perform better than the other types.

## 2. Experimental Procedures

### 2.1. Coating Deposition and Characterization Analyses

As indicated in the introduction, a previous investigation done by the authors has shown that tool life is significantly improved by the application of a TiB_2_ coating [2]. Thus, the same TiB_2_ coating used in that investigation was selected for the current investigation (Coating A). The two other coatings chosen for this investigation (Coating B and Coating C) were the commercial TiB_2_ coatings manufactured by Kennametal (designated as KC5410 grade). All the coating deposition conditions except deposition time were kept constant for Coating B and Coating C. The coating deposition times for Coating B and Coating C were changed to achieve varied coating thicknesses. Coating B had a similar thickness to Coating A, and Coating C was thicker. The coatings had different hardness levels, with Coating A having the lowest hardness. Table 1 highlights the nomenclature for the coatings, including the thickness, hardness, and other micromechanical properties of the coatings.

The coatings were deposited on Kennametal CNMP432 and polished Sandvik Coromant SPGN120308 tungsten carbide inserts in a 6% cobalt matrix. Turning experiments were conducted with the Kennametal CNMP432 inserts. All coating characterizations were performed on the flat, polished Sandvik Coromant SPGN120308 inserts for accurate measurements and ease of use. Coating thicknesses after deposition were measured using the Calotest method with a 25 mm steel ball on SPGN120308 flat inserts. Coating thicknesses were verified with high resolution Scanning Electron Microscope (SEM) images of Focused Ion Beam (FIB) cross sections with a Zeiss NVision 40 (Carl Zeiss, Oberkochen, Germany) dual-beam FIB-SEM instrument. The thicknesses of Coatings A, B, and C were 1.79, 1.82, and 2.89 µm, respectively (Table 1). X-ray diffraction analyses using Cu-Kα (1.544427 Angstroms) radiation were performed on a Proto AXRD Powder Diffraction System (Proto Manufacturing Limited, LaSalle, ON, Canada) to determine the crystal structure and the preferred orientation of the TiB_2_ coatings. Residual stresses in the coatings were calculated by a Proto LXRD Stress Analyzer (Proto Manufacturing Limited, LaSalle, ON, Canada), using the sin^2^ ψ method. The measurements were performed with a multiple exposure technique at 11 ψ (psi) tilts, using a 2.0 mm round aperture with a collection time of 80 s for each diffraction peak. To carry out the measurements, Ti-Kα (2.7497 Angstroms) radiation was used on the (101) plane at a diffraction angle of around 84°. For diffraction peak fitting, a Gaussian function was applied.

X-ray Photoelectron Spectroscopy (XPS) measurements were performed on worn tools (after approximately 100 m cut) to assess the tribo-film formation in a SPECS system (Berlin, Germany) equipped with a Phoibos 150 1D-DLD analyzer and monochromatic radiation source Al Kα (1486.7 eV). An initial analysis was carried out to determine the elements present (wide scan: step energy 1 eV, dwell time 0.1 s, pass energy 80 eV) and detailed analyses of the detected elements were carried out (detail scan: step energy 0.08 eV, dwell time 0.1 s, pass energy 30 eV) with an electron exit angle of 90°. The spectrometer was previously calibrated with Ag (Ag 3d5/2, 368.26 eV). The spectra collected were adjusted using the CasaXPS 2.3.16 software using a Gaussian–Lorentzian function after background subtraction. The concentrations were calculated by correcting the values with relative atomic sensitivity factors.

A Micro Materials NanoTest P3 (Micro Materials, Wrexham, UK) system was used to measure the hardness and elastic modulus of the coatings deposited on the polished WC-Co samples. A Berkovich diamond indenter was used to perform the nanoindentation at room temperature. The indenter was calibrated for indenter shape, load, displacement, and frame compliance according to ISO14577-4 [24]. A minimum of 40 indentations were done at a load of 50 mN. The load was adjusted to diminish the effect of the samples’ surface roughness and prevent the substrate effect by keeping the penetration depth during indentation to less than 10% of the overall coating thickness. Thermal drift correction was done by collecting 60 s of post-indentation data after each indentation. Nano-impact testing was also conducted at room temperature with the P3 system. Five tests were carried out at various locations for each coating with a cube-corner indenter. A load of 20 mN was used to do the impact test after every 4 s for a total test time of 300 s. An Anton Paar-NHT3 Nanoindentation Tester (Buchs, Switzerland) was used to measure the yield stress of the coatings. The test was conducted in the sinus mode at a sinus frequency of 5 Hz and amplitude of 20 mN. A 20 μm end radii spherical diamond indenter was used to analyze the yield property at a load of 200 mN. Twenty tests were carried out at different locations for each coated tool at a loading and unloading rate of 400 mN/min.

An Anton Paar-RST3 Revetest^®^ Scratch Tester (Buchs, Switzerland) was used to perform micro-scratch tests, multi-pass wear tests, and toughness measurements on all coatings. Micro-scratch tests were carried out to study the behavior of the coatings under progressive loading. The tests were conducted with progressive loading from 0.5 N to 5 N over a scratch length of 0.5 mm at a scratching speed of 0.78 mm/min and loading rate of 7.02 N/min. The pre- and post-topography scans were done at a 0.5 N load. A 20 μm end radii Rockwell diamond indenter was used to perform all tests. Three scratch tests were performed on each coating. Multi-pass wear tests were also conducted with the same 20 μm end radii Rockwell diamond indenter. Each test was performed with 5 passes over a track length of 1 mm at a constant load of 0.75 N. Based on the ISO 28,079 standard [25], a modified Palmqvist toughness measurement method was used to calculate the toughness of the coatings. The test was done at a 150 N load with a Vickers diamond indenter. The toughness values were calculated using the ratio of the load to the sum of the total crack lengths at the indentation corners. The images of the imprints were taken with a Vega 3-TESCAN scanning electron microscope (SEM) (Brno Kohoutovice, Czech Republic).

The surface topography and morphology of the TiB_2_ coatings were assessed using the tapping mode of an Anton Parr Tosca^TM^ 400 atomic force microscope (AFM) (Graz, Austria). Commercial silicon probes with 285 kHz of resonant frequency and a force constant of 42 N/m were used to perform the scans. A scan size area of 1 μm × 1 μm was used. Tosca^TM^ analysis software (Version-7.4.8341) was used for image processing and data analysis of the scans. The thermal diffusivity and thermal conductivity of the coatings were measured using the laser flash method with a Discovery Laser Flash DLF- 1200 system (TA Instruments, New Castle, DE, USA). The measurements were done at 100°C increments up to 800 °C.

### 2.2. Experimental Setup and Tool Wear Analyses

Cutting tests were performed to analyze the wear behavior of the coated tools. Wet turning experiments were conducted on an ASTM B265 Grade 5 Ti6Al4V alloy and done on a Nakamura Tome SC450 CNC turning machining center (Nakamura-Tome Precision Industry Co., Ltd., Hakusan, Ishikawa, Japan). The wet turning tests were carried out for roughing operations with industry recommended cutting conditions, as stated in Table 2. Coated CNMP432 carbide grade (WC, 6% Co) K313 Kennametal turning inserts put on a MCLN-5° KenlocTM tool holder were used for all tests. The tool holder was placed on a Kistler Type 9129AA 3-component piezoelectric dynamometer for measuring the resultant cutting forces during machining. The dynamometer was connected to a Kistler type 5010 charge amplifier. The analog to digital signal conversion was done by a National Instruments (NI) Type 9215 data acquisition card in a National Instruments (NI) cDAQ-9172 DAQ system. A 5 kHz sampling rate was used for collecting the data. The data were processed using National Instruments (NI) LabVIEW 2014. All cutting tests were repeated 3 times for each coating to assess repeatability.

Tool life was evaluated in terms of tool flank wear which was measured after each cutting pass using a VHX-5000 Keyence optical microscope (KEYENCE corporation of America, Itasca, IL, USA). The cutting tests were continued until each tool reached a maximum flank wear of 300 µm in accordance with the ISO 3685:1993 standard [26]. The scatter of the flank wear measurements was approximately 10%. Chip morphology analyses were conducted on chips collected after approximately 50 m of cutting using standard practices [27].

Volumetric wear measurements were conducted for all the worn tools after approximately every 600 m of cutting length. The analyses were performed using the focus-variation technology of the Alicona Infinite Focus G5 3D surface measurement system (Alicona Manufacturing Inc., Bartlett, IL, USA). The system is capable of generating 3D topographic images of the cutting tools and of measuring the volumetric difference between the new and worn tools using the Measure Suite module. Before any cutting tests were performed, a 3D volume dataset of the cutting edge of the new tool was collected for reference. It was then compared against the 3D volume dataset of the same cutting edge of the worn tool after approximately every 600 m of cutting length. The Measure Suite module aligned the two datasets and measured the crater wear and built-up volume of the cutting tools by calculating the volumetric difference of the worn tool above and below the reference dataset. The cutting-edge radius of each tool was also measured using the Edge Master module of the Alicona system.

## 3. Results and Discussions

### 3.1. Machining Performance Analysis

**Figure 1:**
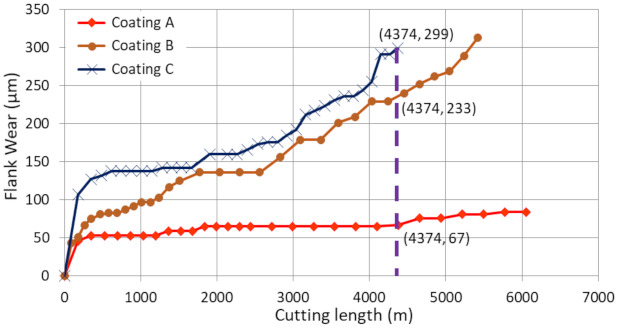
Flank wear vs. cutting length data for the three TiB_2_ coatings during wet rough turning of Ti6Al4V alloy.

To assess the performance of the coatings during the wet rough turning of Ti6Al4V, tool life studies were performed. Figure 1 presents the progression of tool flank wear on the coated tools during machining with respect to cutting length. The highest flank wear intensity was seen for Coating C, followed by Coating B and Coating A. Coating A had approximately 346% tool life improvement compared to Coating C and approximately 248% tool life improvement compared to Coating B. Coating A also showed the lowest BUE intensity.

**Figure 2:**
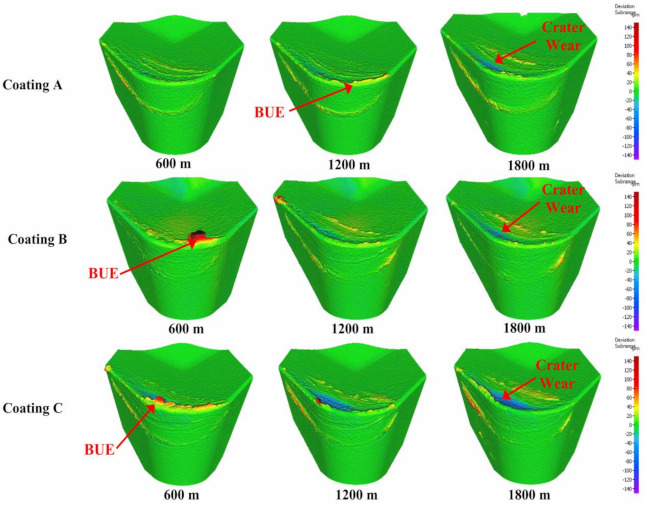
3D progressive wear difference measurement of the coated tools highlighting evolution of built−up layer and crater wear during machining.

**Figure 3:**
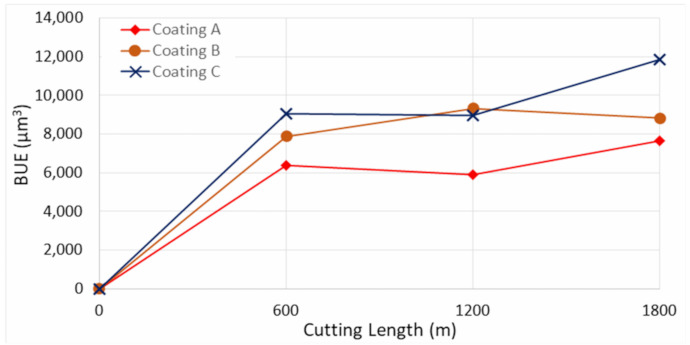
Built up volume progression vs length of cut for the three TiB_2_ coated tools considering the peaks above reference surface of the original tool.

**Figure 4:**
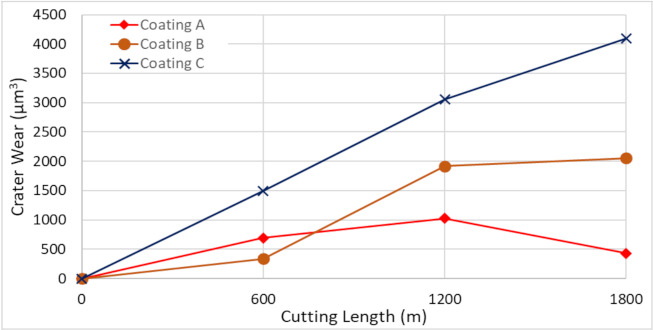
Crater wear volume progression vs length of cut for the three TiB_2_ coated tools considering the peaks below reference surface of the original tool.

Figure 2 shows the progression of tool wear and the 3D difference measurement of the cutting edge after every 600 m of cutting length for the three TiB_2_ coated tools. The tool wear was mainly concentrated on the cutting edge and dictated by two tool wear mechanisms: adhesion of the workpiece material leading to BUE formation at the cutting edge of the tool and crater wear occurring due to the generation of high cutting temperatures. As can be seen from the 3D images, the volume of BUE formation was significantly different for the various coated tools. The harder Coatings B and C showed substantial BUE formation, especially during the initial stages of the cut. Crater wear was also seen to be higher for Coatings B and C. The lowest BUE formation and crater wear progression was observed for Coating A, which had low hardness. Figure 3 and Figure 4 illustrate the evolution of BUE formation and crater wear as cutting progressed for the three TiB_2_ coatings. BUE formation is unstable and dynamic in nature. This behavior is quite evident from the fluctuation in peak volume compared to previous machining passes, as seen in Figure 3. As BUE breaks off, it often leads to coating delamination and tool edge chipping. Coating A’s lower BUE formation and lower progressive build-up while cutting therefore reduced the probability of tool edge chipping. Thus, the reduced BUE formation together with delayed and lower crater wear intensity led to uniform and stable tool wear for Coating A. It is important to point out here that the reduced crater wear value for Coating B at 600 m of cutting length (Figure 4) was due to the BUE covering the crater wear on the rake surface of the tool. This can be corroborated by the 3D images and BUE progressive volume data (Figure 2 and Figure 3). 

**Figure 5:**
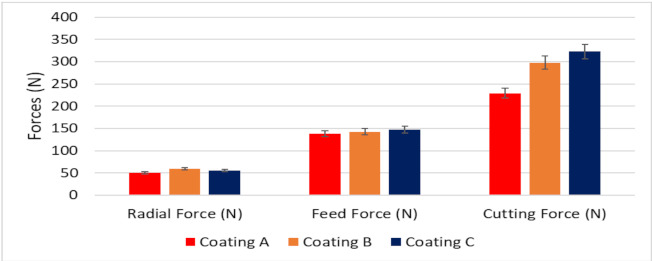
Variation of cutting forces for the three TiB_2_ coated tools.

Figure 5 shows the variation in cutting forces for the three coated tools. The forces in all directions are lower for Coating A than for the others. This indicates that Coating A had lower cutting and frictional forces. All the coated tools had a similar cutting-edge radius (see Table 1) confirming that the variation in cutting forces was entirely due to the coating variations and not due to microgeometry changes in the tool. Chip characteristics provide valuable insights into the tribological interactions taking place at the tool-chip interface during machining. The characteristics of the chips collected after approximately 50 m of cutting length are presented in Table 3. Analysis of the chip characteristics showed an improvement in tribological behavior for Coating A. Both the chip compression ratio and shear plane angle were higher for Coating A. These suggest that the cutting and frictional forces at the tool-chip interface were lower due to lower shearing forces acting on the chips. This complements the data on cutting forces. Coating A also had a higher chip sliding velocity which confirms that there was lower friction at the tool-chip interface and indicates that the tool-chip contact length was shorter. These led to lower cutting zone temperatures and consequently increased the tool life of Coating A.

### 3.2. Coating Characterization

**Figure 6:**
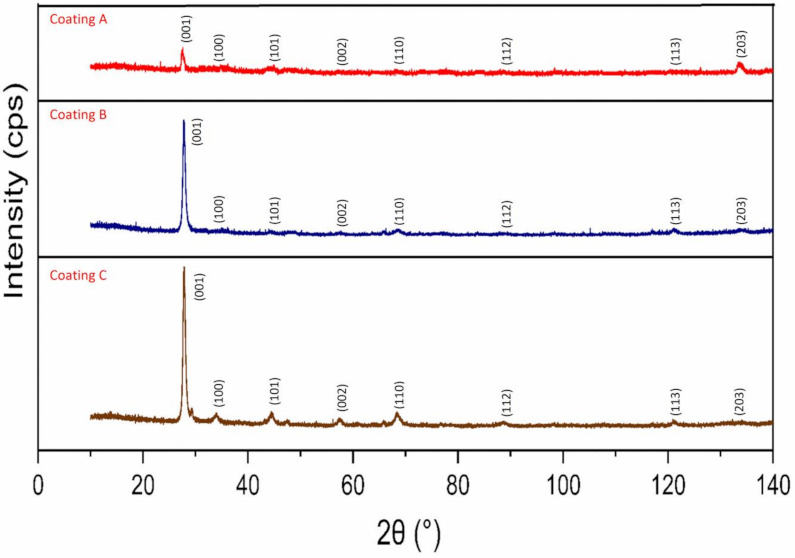
X-ray diffraction patterns of the three TiB_2_ coatings.

Detailed micro-mechanical and structural analyses of the coatings were conducted to assess the wear behavior of the coatings during machining. XRD patterns for all the coatings are shown in Figure 6. These indicate that the crystal orientations and structures of all the TiB_2_ coatings were quite similar. Distinct peaks with varying intensities were observed for the coatings at crystallographic planes of (001), (100), (101), (002), and (110). For all the coatings, the (001) peak had the strongest intensity, suggesting that the coatings had a significant preferred orientation at the (001) crystallographic plane. The XRD analysis shows that all the deposited monolayer TiB_2_ coatings have hexagonal crystal system (ICCD 00-035-0741). The irradiated area for the XRD analysis was the same for all three coatings. The XRD peaks are broader for coating B and C compared to Coating A suggesting higher dislocation density, which is contributing to the higher hardness of Coating B and C.

**Figure 7:**
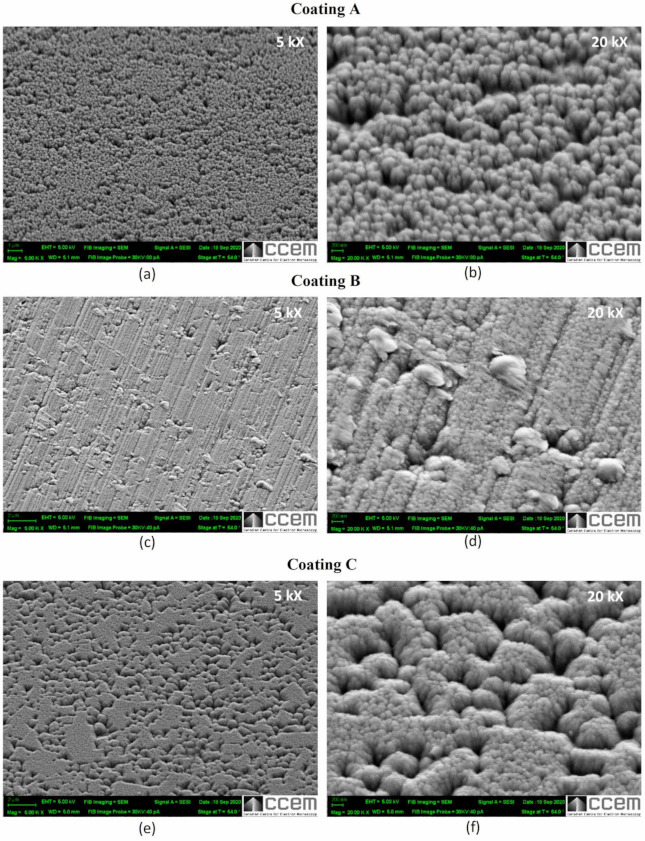
SEM surface morphology of the three TiB_2_ coatings: (**a**,**c**,**e**) at low magnification, (**b**,**d**,**f**) at high magnification.

**Figure 8:**
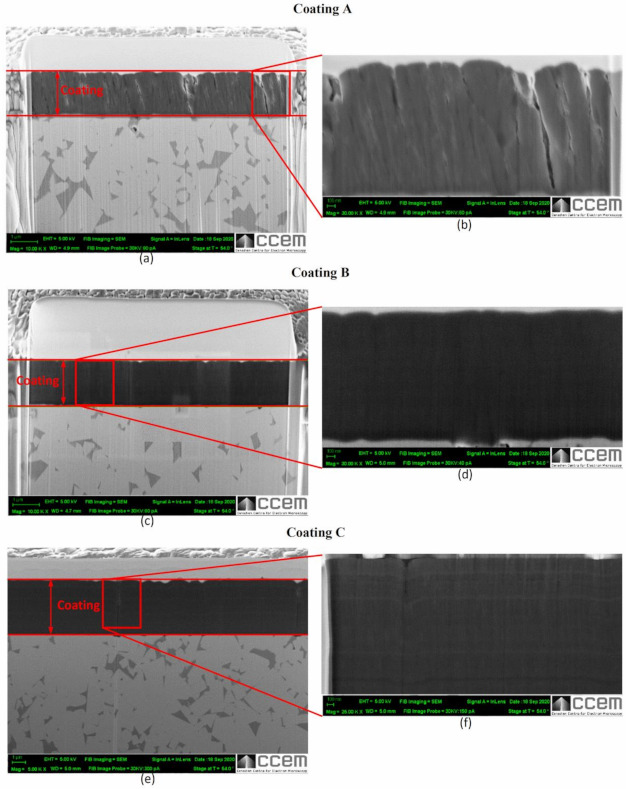
SEM images of FIB cross-sections of the three TiB_2_ coatings showing coating thickness and structure: (**a**,**c**,**e**) at low magnification, (**b**,**d**,**f**) at high magnification.

**Figure 9:**
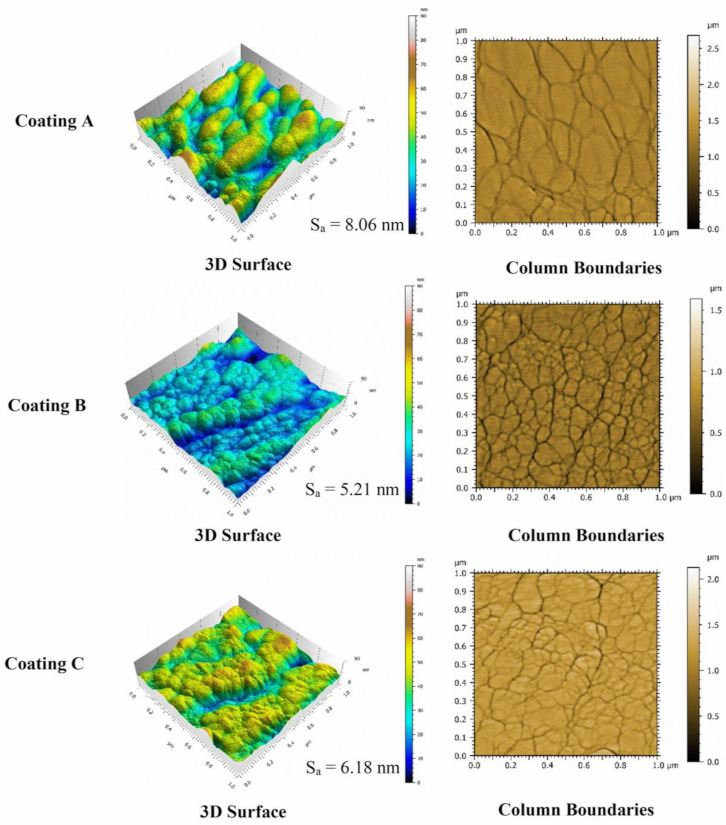
AFM images of surface topography and column boundaries of the different TiB_2_ coatings with arithmetic mean height (Sa) values.

Figure 7 shows high resolution SEM images of the structural characteristics and Figure 8 shows the cross sections of all three coatings. Both figures indicate that all three coatings have columnar structures. The AFM topography scans presented in Figure 9 also show that the size of the top of the columns [28,29] which in case of Coating A is the largest indicating the presence of thicker columns compared to Coatings B and C where the columns are found to be finer. Coating A also shows slightly increased roughness (Sa = 8.06 nm) in nanometer scale due to somewhat more curved column tops [28] compared to coating B (Sa = 5.21 nm) and C (Sa = 6.18 nm). Due to the thickness difference, although still much finer, Coating C has slightly larger column size than Coating B. The effects of these differences in the coating structures are also evident through the differences in their properties and deformation behaviors, as discussed below.

Figure 7, Figure 8 and Figure 9 also show that the column sizes, distributions, and inter-column spacings are different specially in case of Coating A. Previous in-depth investigations [28,29,30,31,32] have shown that these differences in columnar structures can affect coating properties and depending on the application, their performances. For example, inter-column spacings play an important role in effecting residual stress, hardness [28,30,31] and crack propagation [30]. Lower inter-column spacings or highly dense column boundaries result in higher compressive stress and hardness [28]. Therefore, the lower residual stress as well as the hardness (Table 1) in Coating A can be associated with the sub-dense column boundaries or higher inter-column spacings [28] as can be seen in the SEM images. XRD investigations, as discussed earlier, also indicated higher dislocation densities in Coatings B and C which can also contribute to their higher hardness values (Table 1).

The coating thicknesses, micro-mechanical properties, and other related characteristics of the coatings are presented in Table 1. Coating A coating had considerably low hardness compared to the other coatings. The elastic modulus of Coating A was also lower compared to the other two coatings but not as significantly lower as in the case of its hardness. The softer Coating A had lower H/E and H^3^/E^2^ ratios than the other harder TiB_2_ coatings. The H/E and H^3^/E^2^ ratios of the coatings are calculated from the coating’s hardness and elastic modulus. H/E is related to the elastic strain to failure and correlates well with how elastic the contact point of a coating stays during mechanical contacts [33,34]. The H^3^/E^2^ ratio indicates resistance to plastic deformation; a higher value of H^3^/E^2^ usually suggests that the likelihood of coatings to deform plastically is reduced [35]. The substantially lower hardness of Coating A was the main contributing factor for the coating’s lower H/E and H^3^/E^2^ ratio. Such a combination of properties implies that Coating A was less brittle. Although Leyland et al. [33] suggest that coatings with a higher H/E ratio usually led to a reduction in wear, it is reported in the literature that for machining applications with an adhesive wear mechanism, as observed in the rough turning of Ti6Al4V, coatings with lower H/E ratios show better wear resistance [36,37]. Similar observations were also made in the current investigation, where Coating A showed the best wear resistance properties. Hence, for high tool load applications, typical when machining titanium, where severe deformation of the surface layers is possible, an optimized combination of H and E values is crucial for longer tool life.

**Figure 10:**
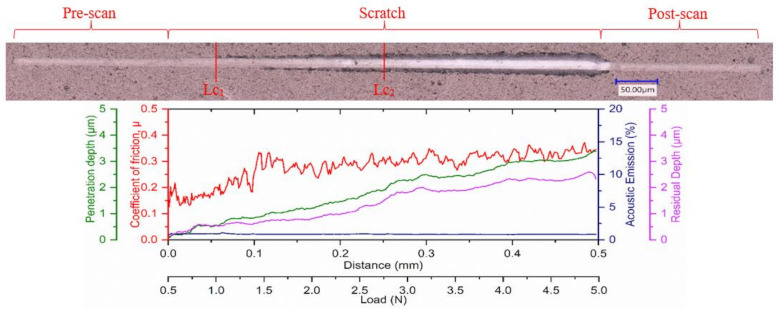
Ramped load scratch test data with optical images of the scratch track for Coating A.

**Figure 11:**
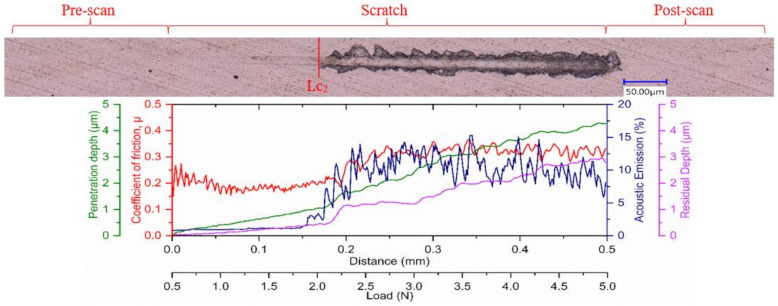
Ramped load scratch test data with optical images of the scratch track for Coating B.

**Figure 12:**
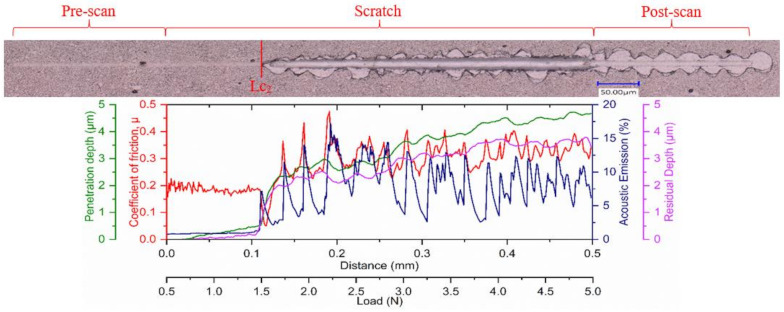
Ramped load scratch test data with optical images of the scratch track for Coating C.

**Figure 13:**
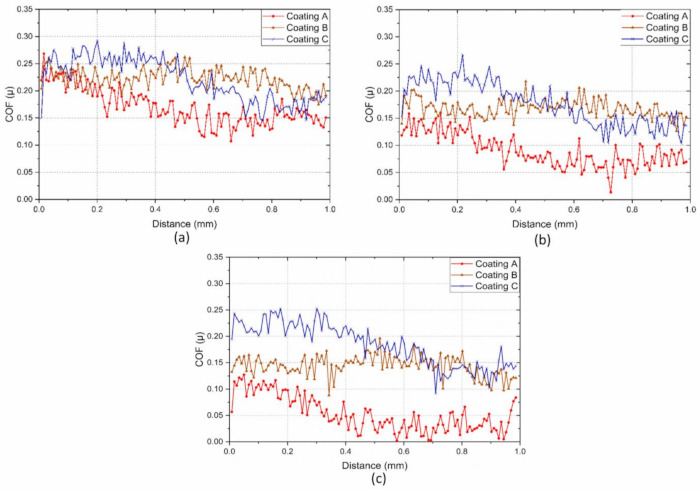
Evolution of coefficient of friction at different passes during repetitive wear test at 0.75 N for the three TiB_2_ coatings after: (**a**) 1 pass, (**b**) 3 passes and (**c**) 5 passes.

It is also important for the applied coating in such applications to have an enhanced ability to dissipate the frictional heat energy that is produced due to intensive deformation of the surface layers. The more energy is dissipated, the less energy is absorbed into the tool effectively reducing deformation and damage to the tool substrate. The plasticity index, which is given by the ratio between the plastic work done and the total work done (plastic and elastic) during indentation [38], can be used to assess a coating’s ability to dissipate energy. Beake et al. [34] reported that a higher plasticity index improved the cutting behavior in turning processes where adhesive wear dominates due to a greater ability to dissipate energy. Thus, a higher plasticity index, in this case for Coating A, indicates the coating’s capability to dissipate higher energy when loaded [2,39]. Higher plasticity of a coating means that the coating releases absorbed energy through plastic deformation and not by brittle fracture, and higher H/E values result in higher critical loads for non-elastic deformation or the onset of yield during indentation or scratch testing [34]. The yield stress, determined from the indentation test, of Coating A (Table 1) was lower and therefore expected to yield early followed by a gradual plastic deformation. On the other hand, the higher yield stress of the harder Coatings B and C suggest that they could withstand higher stress but was followed by drastic brittle deformations. The residual stress data (Table 1) also support this fact, where higher compressive stress in the case of Coatings B and C may have delayed the yield a little, but the inherent higher stress was expected to cause drastic failure. This behavior is also evident in the scratch test results (Figure 10, Figure 11 and Figure 12, Table 4), where the scratch track for Coating A began to show signs of initial deformation or start of cohesive failure at a lower critical load (LC_1_) but underwent plastic deformation without significant cracking and with more localized deformation (i.e., the only substrate exposed is within the scratch track, Figure 10), and the adhesive failure LC_2_ (based on penetration depth data and scratch track analysis) was somewhat delayed compared to the other two. On the other hand, Coatings B and C did not show any gradual deformation or cohesive failure with clear LC_1_ and started to deform drastically in a brittle manner wherever the substrate was exposed, even outside of the scratch track (Figure 11 and Figure 12). The acoustic emission data (Figure 11 and Figure 12) also show clearly how drastic the deformation behavior of Coatings B and C was compared to Coating A. Coating C underwent the worst deformation, indicating inferior tool performance (Figure 1). From a cutting tool life perspective, it could be highly beneficial that the softer Coating A does not chip in the same way as the harder Coatings B and C and less substrate is exposed when the coating fails. In this way, the surface is better protected by the coating layer. Therefore, the scratch test results, coupled with the micro-mechanical properties summarized in Table 1, help to explain why flaking off during cutting was avoided in the softer Coating A layer and why this coating has a potential for better tribological performance under operation. To study the frictional behavior of the coatings, multi-pass constant load scratch tests, also known as wear tests, were performed at a subcritical load of 0.75 N. Figure 13 shows that for all the passes, the coefficient of friction was comparatively lower for Coating A and decreased slightly as the number of passes increased. This indicates that there was less friction at the cutting interface for Coating A than for the other two. A chip characteristics analysis also indicated improved tribological behavior for Coating A (Table 3).

**Figure 14:**
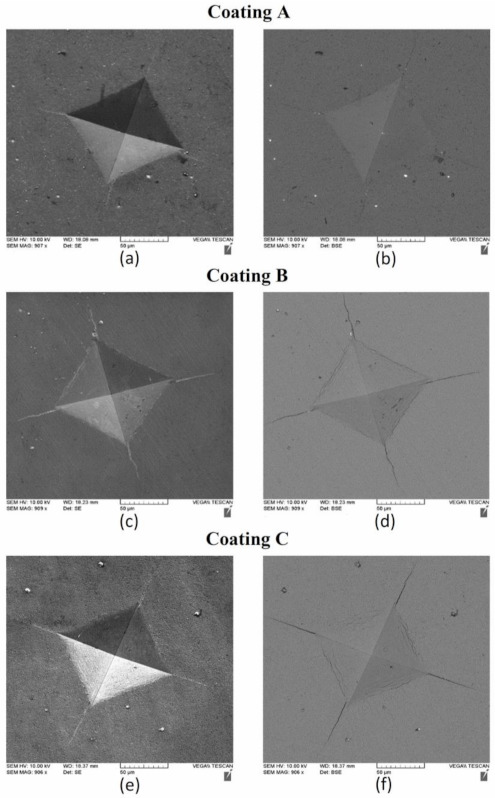
SEM images of Vickers indentation and cracks of the three TiB_2_ coatings from modified Palmqvist toughness test: (**a**,**c**,**e**) SE images, (**b**,**d**,**f**) BSE images.

**Figure 15:**
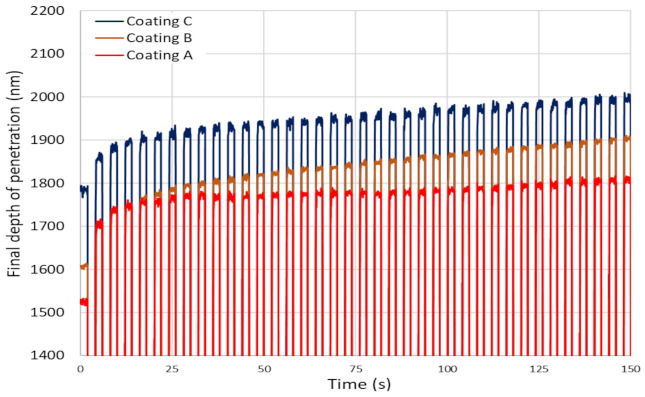
First 150 s depth of penetration obtained from nano-impact testing at a load of 20 mN for the TiB_2_ coatings.

**Figure 16:**
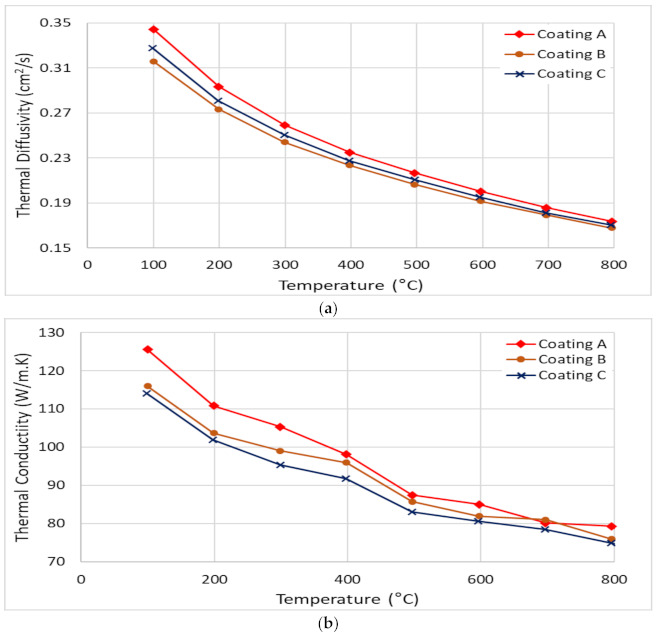
Temperature dependent data of (**a**) thermal diffusivity and (**b**) thermal conductivity for the three TiB_2_ coatings.

It has been previously shown that a higher plasticity index (as seen for Coating A) is indicative of higher toughness and durability of the coatings [40]. Toughness refers to a coating’s ability to absorb energy during deformation without fracturing and to resist crack propagation in the coating [41]. The modified Palmqvist toughness test confirmed that the softer Coating A had higher toughness than the harder Coatings B and C (Table 1). Since all the coatings were deposited on the same substrate material, the variations in the toughness values obtained were entirely due to the applied coatings. Figure 14 shows SEM images of the imprints and the crack formations under indentation load. Cracks along the edges of Coatings B and C indicate the brittleness of these high hardness coatings compared to the softer Coating A. Nano-impact tests were carried out to study the coatings’ fracture resistance under repetitive impact at the same spot with a 20 mN load every 4 s (Figure 15). The evolution of the impact penetration depths due to progressive coating damage was continuously monitored. The increase in penetration depth with repetitive impact was lowest for Coating A, indicating better fatigue fracture resistance. For Coatings B and C, the increase in penetration depth was significantly higher, indicating the inferior performance of these two coatings. Thus, the higher plasticity index, lower H/E ratio, higher modified Palmqvist toughness, and improved fatigue fracture resistance of the softer Coating A indicate that this coating has the ability to withstand more plastic deformation than the other two which, from the higher hardness values and higher H/E ratio, are more likely to fail in a brittle manner. For machining applications where an adhesive wear mechanism leading to BUE formation dictates tool wear, the coating properties that Coating A possesses are crucial. BUE is an unstable structure that breaks off intermittently during machining causing surface damage and coating delamination [42]. Coatings that fail in a brittle manner are more prone to coating delamination during BUE removal. Moreover, Coating A’s increased thermal diffusivity and conductivity even at higher temperatures in comparison with the other two, as shown in Figure 16, will be beneficial for thermal energy dissipation at the cutting zone, saving the tool from overheating. This also ensures that Coating A performs better, resulting in longer tool life (Figure 1).

**Figure 17:**
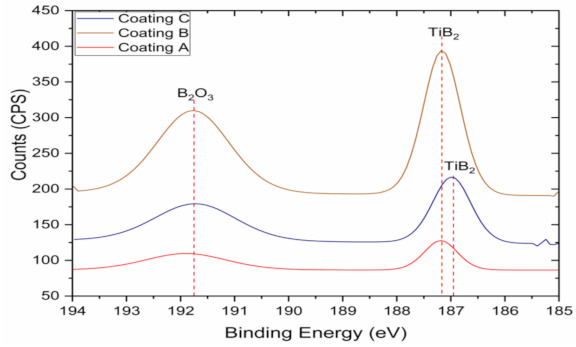
High resolution XPS data (B 1s spectrum) of worn rake surface of the three TiB_2_ coatings confirming B_2_O_3_ tribo-oxide formation.

XPS analysis on the worn tools showed B_2_O_3_ tribo-film formation (Figure 17). Table 5 shows the relative percentages of the tribo-films for the three TiB_2_ coatings. These tribo-films form due to the self-lubricating nature of the TiB_2_ coatings [2,22] and enhances lubriciousness of the coating thereby reducing friction at the tool-chip interface. As seen from Table 5, Coating A actually has the least amount of tribo-film formation although it gave the best tool performance (Figure 1). This suggests that the superior machining performance of Coating A is mostly attributed to its unique and superior micromechanical and tribological properties, not just due to the tribo-film formation. Therefore, Coating A, with a higher toughness and plasticity index combined with lower hardness, lower yield stress, lower H/E and H^3^/E^2^ ratios, and improved thermal properties, provides better tool protection and longer tool life by preventing coating delamination during cutting.

## 4. Conclusions

Tool performance during the rough turning of Ti6Al4V alloy is mainly dictated by an adhesive wear mechanism leading to significant BUE formation. An effective strategy to improve tool performance in such cases is to use a lubricious tool coating that has its mechanical and adaptive characteristics tweaked to provide better tribological interaction at the tool-chip interface and reduce and minimize the effects of BUE formation. Amongst others, the TiB_2_ family of coatings have shown great promise for the machining of titanium alloys.

The micromechanical and tribological properties of a coating have a direct relation to the wear performance of a tool under machining conditions associated with strong built-up edge formation. The properties of the coating strongly vary depending on the deposition parameters and techniques applied. In the current research, three TiB_2_ coatings deposited by magnetron sputtering with varied coating thicknesses and hardness values were investigated. All the three TiB_2_ coatings formed B_2_O_3_ tribo-films during machining which increased lubriciousness of the coatings reducing friction at the tool-chip interface. However, despite having lower amount of tribofilm formation, the thin TiB_2_ coating with low hardness showed more significant tool life improvement during the rough turning of Ti6Al4V alloy than the thin and thick TiB_2_ coatings with high hardness. This could be attributed to the fact that the low hardness TiB_2_ coating has the ability to minimize BUE formation and prevent coating delamination due to its unique blend of micromechanical and tribological properties. The low hardness TiB_2_ coating under the tested machining conditions presents favorable combinations of mechanical and thermal properties and improved tribological properties with less substrate exposure, which ultimately leads to enhanced tool performance.

## Figures and Tables

**Table 1:** Micro-mechanical properties of the three monolayer TiB_2_ coatings.

Nomenclature	Coating A	Coating B	Coating C
Thickness, µm	1.79	1.82	2.89
Hardness (H), GPa	9.49 ± 0.516	36.03 ± 2.156	34.18 ± 3.512
Elastic Modulus (E), GPa	413.51 ± 10.56	582.12 ± 11.50	581.14 ± 24.33
Plasticity Index	0.778	0.494	0.507
H/E ratio	0.023	0.062	0.059
H^3^/E^2^ ratio	0.005	0.138	0.118
Cutting edge radius of coated tools, µm	51.38 ± 3.17	49.77 ± 3.58	53.01 ± 5.20
Modified Palmqvist Toughness, N/µm	1.081	0.897	0.643
Yield Stress, MPa	4600	8007	7936
Residual Stress, MPa	−633 ± 53	−842 ± 79	−930 ± 36

**Table 2:** Cutting parameters for machining of Ti6Al4V alloy.

Machining Operation	Cutting Tool Substrates	Workpiece Material	Workpiece Hardness, HRC	Cutting Speed, m/min	Feed, mm/rev	Depth of Cut, mm	Coolant Condition
Rough Turning	Kennametal CNMP432 Grade K 313 Turning inserts	ASTM B265 Grade 5 Ti6Al4V alloy	37–38	45	0.15	2	Flood coolant (Xtreme Cut 290)

**Table 3:** Tribological performance evaluated through chip characteristics for the TiB_2_ coatings.

Coating	Chip Compression Ratio—CCR	Φ—Shear Angle (°)	(ϒ) Shear Strain	Chip Sliding Velocity (m/min)
Coating A	1.18	53.7	0.50	52.9
Coating B	1.03	49.6	0.63	46.5
Coating C	1.07	50.7	0.59	48.2

**Table 4:** Critical load for crack initiation in coating, LC_1_ and critical load for coating failure, LC_2_ obtained from ramped load scratch test for the three TiB_2_ coatings.

Coating	LC_1_, N	LC_2_, N
Coating A	1.00	2.75
Coating B	-	2.01
Coating C	-	1.50

**Table 5:** Relative percentages of the B_2_O_3_ phase present on the rake surface of the three TiB_2_ coated tools after approximately 100 m cut obtained from High Resolution XPS.

Coating	Binding Energy (eV)	Relative Atomic Percentage (%)
Coating A	191.9	2.348
Coating B	191.8	4.56
Coating C	191.7	3.815

## Data Availability

All data are presented in the current manuscript. For further query, communicate with the corresponding author.

## References

[1] Veiga C., Davim J.P., Loureiro A.J.R. (2012). Proprties and Applications of Titanium Alloys. Rev. Adv. Mater. Sci..

[2] Chowdhury M., Chowdhury S., Yamamoto K., Beake B., Bose B., Elfizy A., Cavelli D., Dosbaeva G., Aramesh M., Fox-Rabinovich G. (2017). Wear behaviour of coated carbide tools during machining of Ti6Al4V aerospace alloy associated with strong built up edge formation. Surf. Coatings Technol..

[3] Paiva J.M., Shalaby M.A.M., Chowdhury M., Shuster L., Chertovskikh S., Covelli D., Junior E.L., Stolf P., Elfizy A., Bork C.A.S. (2017). Tribological and Wear Performance of Carbide Tools with TiB_2_ PVD Coating under Varying Machining Conditions of TiAl6V4 Aerospace Alloy. Coatings.

[4] Dearnley P., Grearson A. (1986). Evaluation of principal wear mechanisms of cemented carbides and ceramics used for machining titanium alloy IMI 318. Mater. Sci. Technol..

[5] Boyer R. (1996). An overview on the use of titanium in the aerospace industry. Mater. Sci. Eng. A.

[6] Armendia M., Garay A., Iriarte L.-M., Arrazola P.J. (2010). Comparison of the machinabilities of Ti6Al4V and TIMETAL® 54M using uncoated WC–Co tools. J. Mater. Process. Technol..

[7] Corduan N., Himbart T., Poulachon G., Dessoly M., Lambertin M., Vigneau J., Payoux B. (2003). Wear Mechanisms of New Tool Materials for Ti-6AI-4V High Performance Machining. CIRP Ann..

[8] Hartung P.D., Kramer B.M. (1982). Tool Wear in Machining Titanium. Ann. CIRP.

[9] Biksa A., Yamamoto K., Dosbaeva G., Veldhuis S., Fox-Rabinovich G., Elfizy A., Wagg T., Shuster L. (2010). Wear behavior of adaptive nano-multilayered AlTiN/MexN PVD coatings during machining of aerospace alloys. Tribol. Int..

[10] M’Saoubi R., Axinte D., Soo S.L., Nobel C., Attia H., Kappmeyer G., Engin S., Sim W.-M. (2015). High performance cutting of advanced aerospace alloys and composite materials. CIRP Ann..

[11] Hatt O., Crawforth P., Jackson M. (2017). On the mechanism of tool crater wear during titanium alloy machining. Wear.

[12] Wang Z.M., Ezugwu E.O. (1997). Performance of PVD-Coated Carbide Tools When Machining Ti-6Al-4V©. Tribol. Trans..

[13] Özel T., Sima M., Srivastava A., Kaftanoglu B. (2010). Investigations on the effects of multi-layered coated inserts in machining Ti–6Al–4V alloy with experiments and finite element simulations. CIRP Ann..

[14] Bouzakis K.-D., Michailidis N., Skordaris G., Bouzakis E., Biermann D., M’Saoubi R. (2012). Cutting with coated tools: Coating technologies, characterization methods and performance optimization. CIRP Ann..

[15] Cherukuri R., Molian P. (2003). Lathe Turning of Titanium Using Pulsed Laser Deposited, Ultra-Hard Boride Coatings of Carbide Inserts. Mach. Sci. Technol..

[16] Berger M., Hogmark S. (2002). Evaluation of TiB_2_ coatings in sliding contact against aluminium. Surf. Coatings Technol..

[17] Park B., Jung D.-H., Kim H., Yoo K.-C., Lee J.-J., Joo J. (2005). Adhesion properties of TiB_2_ coatings on nitrided AISI H13 steel. Surf. Coatings Technol..

[18] Silva M.F., Hancock P., Nicholls J. (2000). Multilayer Coating Techniques to Optimise the Properties of TiB_2_-Based Coatings. Adv. Eng. Mater..

[19] Berger M., Larsson M., Hogmark S. (2000). Evaluation of magnetron-sputtered TiB_2_ intended for tribological applications. Surf. Coatings Technol..

[20] Kelesoglu E., Mitterer C. (1998). Structure and properties of TiB_2_ based coatings prepared by unbalanced DC magnetron sputtering. Surf. Coatings Technol..

[21] Grancic B., Mikula M., Hruba L., Gregor M., Stefecka M., Csuba A., Dobročka E., Plecenik A., Kus P. (2005). The influence of deposition parameters on TiB_2_ thin films prepared by DC magnetron sputtering. Vacuum.

[22] Fox-Rabinovich G.S., Gershman I., El Hakim M.A., Shalaby M.A., Krzanowski J.E., Veldhuis S.C. (2014). Tribofilm Formation As a Result of Complex Interaction at the Tool/Chip Interface during Cutting. Lubricants.

[23] Chowdhury M., Bose B., Rawal S., Fox-Rabinovich G., Veldhuis S. (2021). Investigation of the Wear Behavior of PVD Coated Carbide Tools during Ti6Al4V Machining with Intensive Built Up Edge Formation. Coatings.

[24] ISO 14577-4: 2016(E) (2016). Metallic Materials—Instrumented Indentation Test for Hardness and 746 Materials Parameters—Part 4: Test Method for Metallic and Non- Metallic Coatings.

[25] ISO 28079: 2009 (E) (2009). Hardmetals—Palmqvist Toughness Test.

[26] ISO 3685: 1993(E) (1993). Tool-Life Testing with Single-Point Turning Tools.

[27] Shaw M.C., Cookson J. (1985). Metal cutting principles. Tribol. Int..

[28] Luo Q., Lewis D., Hovsepian P., Münz W.-D. (2004). Transmission Electron Microscopy and X-ray Diffraction Investigation of the Microstructure of Nanoscale Multilayer TiAlN/VN Grown by Unbalanced Magnetron Deposition. J. Mater. Res..

[29] Luo Q., Wang S.C., Zhou Z., Chen L. (2011). Structure characterization and tribological study of magnetron sputtered nanocomposite nc-TiAlV(N,C)/a-C coatings. J. Mater. Chem..

[30] Ganvir A., Joshi S., Markocsan N., Vassen R. (2018). Tailoring columnar microstructure of axial suspension plasma sprayed TBCs for superior thermal shock performance. Mater. Des..

[31] Fan Z., Wang K., Dong X., Duan W., Mei X., Wang W., Cui J., Lv J. (2015). Influence of columnar grain microstructure on thermal shock resistance of laser re-melted ZrO2-7wt.% Y2O3 coatings and their failure mechanism. Surf. Coatings Technol..

[32] Qiu S.-Y., Wu C.-W., Huang C.-G., Ma Y., Guo H.-B. (2021). Microstructure Dependence of Effective Thermal Conductivity of EB-PVD TBCs. Materials.

[33] Leyland A., Matthews A. (2000). On the significance of the H/E ratio in wear control: A nanocomposite coating approach to optimised tribological behaviour. Wear.

[34] Beake B., Fox-Rabinovich G. (2014). Progress in high temperature nanomechanical testing of coatings for optimising their performance in high speed machining. Surf. Coat. Technol..

[35] Tsui T.Y., Pharr G.M., Oliver W.C., Bhatia C.S., White R.L., Anders S., Anders A., Brown I.G. (1995). Nanoindentation and Nanoscratching of Hard Carbon Coatings for Magnetic Disks. MRS Proc..

[36] Fox-Rabinovich G., Shuster L., Beake B., Veldhuis S. (2006). Physical and Mechanical Properties to Characterize Tribological Compatibility of Heavily Loaded Tribosystems (HLTS). Self-Organization during Friction: Advanced Surface-Engineered Materials and Systems Design.

[37] Sato K., Ichimiya N., Kondo A., Tanaka Y. (2003). Microstructure and mechanical properties of cathodic arc ion-plated (Al,Ti)N coatings. Surf. Coat. Technol..

[38] Beake B., Fox-Rabinovich G., Veldhuis S., Goodes S. (2009). Coating optimisation for high speed machining with advanced nanomechanical test methods. Surf. Coat. Technol..

[39] Fox-Rabinovich G., Veldhuis S., Scvortsov V., Shuster L.S., Dosbaeva G., Migranov M. (2004). Elastic and plastic work of indentation as a characteristic of wear behavior for cutting tools with nitride PVD coatings. Thin Solid Films.

[40] Zhang X., Beake B.D., Zhang S. (2015). Toughness evaluation of hard coatings and thin films. Thin Films and Coatings: Toughening and Toughness Characterization.

[41] Zhang S., Zhang X. (2012). Toughness evaluation of hard coatings and thin films. Thin Solid Films.

[42] Fox-Rabinovich G., Kovalev A. (2006). Self-Organization and Structural Adaptation during Cutting and Stamping Operations. Self-Organization during Friction: Advanced Surface-Engineered Materials and Systems Design.

